# Dynamic and transient processes in warm dense matter

**DOI:** 10.1098/rsta.2022.0223

**Published:** 2023-08-21

**Authors:** Thomas G. White, Jiayu Dai, David Riley

**Affiliations:** ^1^ Department of Physics, University of Nevada, Reno, NV 89557, USA; ^2^ College of Science, National University of Defense Technology, Changsha 410073, People’s Republic of China; ^3^ School of Mathematics and Physics, Queen’s University Belfast, Belfast BT7 1NN, UK

**Keywords:** warm dense matter, review, plasmas, experimental techniques, simulation

## Abstract

In this paper, we discuss some of the key challenges in the study of time-dependent processes and non-equilibrium behaviour in warm dense matter. We outline some of the basic physics concepts that have underpinned the definition of warm dense matter as a subject area in its own right and then cover, in a selective, non-comprehensive manner, some of the current challenges, pointing along the way to topics covered by the papers presented in this volume.

This article is part of the theme issue ‘Dynamic and transient processes in warm dense matter’.

## 1. Introduction

The term warm dense matter (WDM) was coined over 20 years ago, e.g. [1–3] to describe matter that is at high pressures, generally over 100 GPa, but at the same time, at high temperatures of order $10^{4}\text{–}10^{6}\;K$. This range of conditions leads to partially degenerate and partially ionized matter, with strong correlations between charged particles and both populated excited and bound states playing a role in the microscopic structure and particle dynamics [4]. It is a transitional state between solid and plasma and exhibits properties of both. Its inherent complexities make perturbative techniques unreliable, leading to significant differences in theoretical and computational predictions of important quantities [5]. Moreover, due to the difficulty of creating and diagnosing WDM in the laboratory [6], there is a shortage of experimental results with which to benchmark predictions.

## 2. Background

Much of the physics relevant to WDM was studied in the fields of dense plasma physics [7,8] and liquid metal physics [9] long before the term was coined. The remit of these two fields includes a wider range of conditions than is usually included within the definition of WDM but they do share some issues, such as the effect of degeneracy and particle correlation on basic properties such as thermal and electrical conductivity.

A principal and oft-cited motivation for the study of WDM is that the relevant conditions are applicable to many planetary interiors, especially for large planets such as Jupiter and Saturn. For the former, the expected conditions at the core, depending on the equation of states used, include a pressure of order 4 TPa and temperature approximately $10^{4}\;K$. A key point emerging from work such as that by Nettelmann and colleagues [10] is that, by using different equation of state models for the hydrogen and helium within the planetary core, various models of the interior layers and conditions can be constructed that still agree with observations of surface temperature and gravitational moments observed by missions such as Voyager and Cassini, e.g. [11–13]. The significantly improved gravity data from the Juno mission (see [14–16] and references therein) means that more detailed models of the interior of Jupiter can be considered and compared. For example, the more accurate measurements of the J${\;}_{4}$ and J${\;}_{6}$ moments have suggested a more diluted core with heavy and light element mixing. This means that more exact information on the equation of state for hydrogen and helium has more impact than ever and efforts to improve measurements in the laboratory are more important than ever. These considerations highlight the necessity of laboratory experiments to determine equations of states for the WDM regime. The discovery over recent decades of thousands of exoplanets [17,18] only gives increased motivation in this respect.

Interest is not limited to planetary interiors. For any matter taken on a path from a cold solid to hot plasma, such as components of an inertial confinement fusion (ICF) capsule, the sample must pass through the WDM regime, and thus the properties of WDM may affect the trajectory. The design of ICF implosions depends on the thermal conductivity of both the ablator materials and the fuel mixture [19–21]. At the same time, the efficiency of the process can be reduced by fuel degradation through mechanisms like diffusion [22–24]. The Rayleigh–Taylor instability, which can affect the interface between the ablator and fuel within the fusion capsule, is particularly sensitive to the diffusion coefficient [25]. The recent successes at the National Ignition Facility, e.g. [26] can only serve to further increase interest in WDM research within the ICF community.

**Figure 1.**
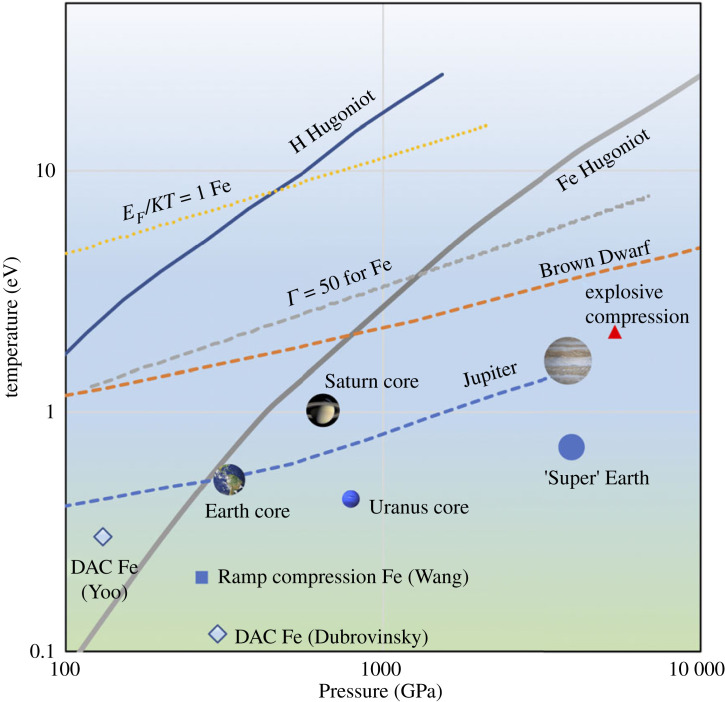
Mapping of the warm dense matter region. The boundaries are a broad guide. We can see the way in which conditions in Jupiter as a function of depth (dashed blue line) pass through the WDM regime, as do conditions in a Brown Dwarf (dashed orange line). We note that static methods of compression and heating, such as diamond anvil cells (DAC), e.g. [27,28] can only probe a peripheral part of the WDM regime. (Adapted from [29].)

In figure 1, we see the WDM regime mapped out in pressure–temperature space. We can see how the shock Hugoniots for two elements of astrophysical interest, hydrogen and iron, pass through the WDM regime. The label, Γ, against the grey dashed curve refers to the ion–ion coupling parameter, which measures the ratio of Coulombic potential energy to thermal energy:

2.1 $$\Gamma =\frac{Z^{2}e^{2}}{ak_{B}T}$$

where Z is the average charge on the ions, and a is a characteristic distance between ions (Wigner–Seitz radius) given by

2.2 $$a={\left(\frac{3}{4\pi n_{i}}\right)}^{1/3}$$

where $n_{i}$ is the average density of the ions. For strong shocks, we typically see values of Γ≲50, and the thermal energy stops being a small perturbation to the Coulomb interaction. The yellow line denotes where the Fermi energy (E${\;}_{F}$) for Fe is equal to the temperature, in eV, indicated in the figure. In this regime, we cannot treat the free electrons in the sample as fully degenerate and we can see that the regime extends across a wide range of pressures and temperatures from a few eV to several 10s of eV.

The significance of planetary interiors as a reason to study WDM means that the conditions we are motivated to study are often in a steady or quasi-steady state with a very long timescale of evolution. For static methods of producing WDM states, Anzellini & Boccato [30] have recently reviewed the accessible range of DACs for both laser and resistive heating. Temperatures of up to 6000 K are reachable for pressures in the region of more than 100 GPa. Tateno *et al.* [31] reported measurements of iron up to 5700 K and 377 GPa, while Dubrovinsky *et al.* [32] report pressures above 750 GPa with a double-stage DAC. Although these extreme conditions are relevant, for example, to the structure of the Earth’s core, as we can see from figure 1, the temperatures reached are at the lower border of the WDM regime and the interiors of larger planets may be out of reach for DAC and other static methods. This presents a challenge for experimental study, and it is for this reason we often look to dynamic methods of production, such as shocks and other rapid heating mechanisms, as discussed in the next section.

## 3. Timescale considerations for dynamic production of warm dense matter

As noted in the previous section, when attempting to access conditions relevant to either planetary interiors or inertial fusion efforts, the appropriate conditions can be well outside the range accessible to static methods. Therefore, to reach conditions within the range of interest, we must turn to dynamic heating and compression methods, which can take several forms. For example, strong shocks (more than 100 GPa) can generate matter at several times solid density and temperatures well in excess of $10{\;}^{4}\;K$ [33,34]. Shocks strong enough to heat a solid beyond this temperature can be generated in a variety of ways; for example, intense lasers of nanosecond duration have commonly been used to generate pressures ranging from 0.1 to 1 TPa [34]. Intense lasers [35,36] or Z-pinch machines [37] can also be used to generate in the range of 100s kJ of X-ray radiation in the sub-keV to keV photon range that can, on timescales of several nanoseconds, volumetrically heat a sample into the WDM region. In a similar vein, intense short-pulse lasers can be used to generate high-intensity electron [38] or proton beams [39] to heat solid targets volumetrically on sub-nanosecond timescales. There are also longer timescale experiments; for example, Gardelle *et al.* used beams of intense electrons from pulsed power generators to generate WDM on 100 ns timescales [40]. Finally, the longest-lived experiments utilize flyer plates driven by explosives in gas-gun experiments that create shocks on microsecond timescales that can reach pressures in excess of 600 GPa [41]. Z-pinches can, in addition to being used to drive X-rays, also be used to accelerate flyer plates to over $40\;{\mathrm{km}\;s}^{-1}$ using the enormous **JXB** forces possible, see [42] for a review of Z-pinch HED science.

Considering the timescale of experiments raises concerns regarding the validity of extrapolating laboratory experiments to conditions of interest, such as those encountered in a planetary core. However, answering this question is not a simple matter, as samples expand upon heating, and the hydrodynamic timescale is dependent on both the pressure and sample size. For example, if we take a sound speed given by

3.1 $$c_{s}\approx \sqrt{\frac{P}{\rho }},$$

for a sample at pressure 100 GPa and density of $10^{3}\;{\mathrm{kgm}}^{-3}$, we can see that the sound speed is of order $10^{4}\;m\;s^{-1}$. For a millimetre-sized sample, the estimated timescale for decompression is $t∼d/c_{s}∼100\;\mathrm{ns}$, where d is the size of the sample. In this sense, we can say that experiments carried out with nanosecond creation and probing can be applicable to steady-state conditions.

However, it is important to note that hydrodynamic expansion timescales are not the only timescale of interest. Not all species in a sample (e.g. ions and electrons) are initially heated in the same way when creating WDM. For example, shock compression heats ions, e.g. [43,44], which transfer energy to electrons through collisions. By contrast, X-ray heating, e.g. [45] transfers energy to electrons via photo-ionization and these transfer energy to ions via collisional processes and electron–phonon coupling. The rate at which energy is transferred between ions and electrons, and indeed the rate at which processes, such as melting, occur, is an important factor to consider when studying the formation of WDM and forms an important topic in this collection addressed by several papers. Not only will this rate determine if we have conditions relevant to equilibrium situations, but macroscopic quantities such as electrical and thermal conduction depend on this rate. These latter quantities are of vital importance in planetary interiors.

Interest in the issue of electron–ion equilibration is further motivated by the fact that short-pulse laser systems have also been used to extend WDM experiments to the other end of the temporal scale, for example, Lecherbourg *et al.* [46] will present results on the evolution of solid copper into a warm dense matter state, in this case, after excitation with a femtosecond laser pulse. More recently, and of particular significance to the work in this issue, the advent of X-ray free-electron lasers has made it possible to achieve volumetric heating on sub-picosecond timescales [45,47]. Using this property, Ren *et al.* [48] discuss the application of inner shell photoionization to creation of a solid density X-ray laser using volumetric pumping with a free electron laser, with particular attention paid to collisional effects of photo-ejected electrons, an issue of relevance to volumetric X-ray heating of WDM. In another paper, Hu *et al.* [49] discuss electron-lattice equilibration within the context of ultra-fast X-ray diagnostics, but again, with physics encompassing processes relevant to WDM production. The development of the field of WDM physics means experimental and modelling techniques progress together, often hand-in-hand. However, for convenience, in the next two sections we present their discussion separately.

## 4. Experimental approaches

It is essential to validate our theoretical knowledge of warm dense matter through experiments, and significant effort has been invested in producing benchmark-quality experimental results. A significant portion of the experimental effort has been dedicated to measuring transport coefficients, including attempts to measure electrical conductivity via the reflectivity of incident electromagnetic radiation from a solid target [50–53]. An early example was the use of a nanosecond optical probe as a diagnostic looking at the time resolved reflectivity history of the rear surface of a shock-compressed foil, e.g. [54] with approximately 10 ps resolution. With the development of chirped pulse amplification (CPA) laser technology in the late 1980s, Strickland & Mourou [55] allowed for much shorter, sub-picosecond pulses. This made it possible to conduct experiments where the reflectivity of the target is measured before any substantial decompression from the well-defined solid density is possible. This can be done for a target heated by the probe itself, e.g. [56] or with a pump-probe arrangement in a relatively simple reflectivity measurement, e.g. [57]. For a more complex arrangement, such as Fourier domain interferometry (FDI), e.g. in [58–60], the motion of the heated surface can be followed and factored into calculations and experimental conclusions.

An important issue closely related to conductivity measurements is the experimental investigation into electron–ion equilibration in the WDM regime. However, hindered by the opacity of WDM, there are limited experimental measurements of the electron–ion equilibration rate in this regime, with only a few model-dependent techniques in use. Historically, the response of the electron subsystem was measured in pump–probe experiments that used optical [57] reflectivity, surface optical emission [44,61] or X-ray absorption spectroscopy [62,63]. Likewise, the bulk ion temperature has only been inferred from the atomic structure, measured through ultrafast electron [64] and X-ray diffraction [38,39,65,66]. For the case of X-ray diagnostics of WDM, the opacity is an important quantity, and Hansen [67] presents a method of calculating non-equilibrium opacity using a DFT-based average atom approach that can be applied across a wide range of conditions including rapidly heated, non-equilibrium samples.

The experiments of Chen *et al.* [57] on warm dense gold revealed an electron–ion coupling parameter that was notably lower than predicted by classical Spitzer theory or the Fermi Golden Rule approach, which allows for electron–ion energy exchange through normal modes of the electron fluid interacting with normal modes of the ions. Instead, it was concluded that coupled modes of the electrons and ions likely play an important role. The observed lower coupling parameter is in line with earlier results from Ng *et al.* [61] and Celliers *et al.* [44], who used temporally resolved measurement of the optical emission from the rear surface of shock-compressed WDM to infer electron-equilibration rates. These studies also indicated that the timescale for electron–ion coupling in strongly coupled plasma was over an order of magnitude longer than expected from equilibrium models such as Spitzer [68] and Lee & More [69], including degeneracy corrections [70], suggesting that equilibration could take hundreds of picoseconds. This has significant implications for shock measurements [44], which commonly use the Rankine–Hugoniot equations to connect shock and particle speeds to density, pressure, and internal energy density in order to assess the equation of state for WDM. These equations rely on accurate temperature measurements to connect to internal energy, which can be affected by equilibration effects and are often obtained using techniques such as streaked optical pyrometry as used by Ng *et al.* [61] and Celliers *et al.* [44].

Many experiments, such as those cited above, are naturally carried out in the optical region where ultra-short pulse pump-probe experiments down to 10 s of femtoseconds duration are available. However, the reflectivity measured then gives access to the ac conductivity. There is much interest in dc conductivity as this is more relevant to understanding WDM in many situations of interest. To this end, there has been a strong development of probing capability using terahertz (THz) radiation, derived from intense ultra-short pulse optical interaction, e.g. [71]. The advantage is that, for a Drude-type model of the conductivity, we can relate the ac conductivity to the dc value via

4.1 $$\sigma (\omega )=\frac{\sigma_{DC}}{1-i\omega \tau }.$$

In reality, of course, there may be complicating factors such as band structure at higher density and lower temperatures. Indeed, non-Drude like behaviour has been reported with DFT calculations of the dynamic conductivity of warm dense Al for energy transfers large enough to cause excitations, e.g. [72].

The significantly lower frequencies in the THz regime mean the initial measurements are closer to the dc values pursued. Despite being complex and difficult to implement, one key advantage of terahertz radiation is that it can penetrate into solid-density samples and, thus, is capable of probing bulk properties of highly excited solid foil samples, e.g. [73].

A more common approach to probing bulk properties of a WDM sample has been to use laser-produced X-rays. Some important early experiments used X-ray Thomson scattering to extract thermodynamic quantities such as temperature and density [74]. However, careful measurement of the plasmon spectrum encodes dynamic information on particle collisions as well as detailed balance [75]. The use of X-ray Thomson scattering to extract thermodynamic properties from experimental data is complex and depends on making approximations in the various theoretical models used. Dornheim *et al.* [76] address this by discussing a new approach based in use of imaginary-time correlation functions. More recently, high-resolution X-ray radiography experiments are being developed with the aim of directly observing the effects of transport properties, such as the thermal conductivity and particle diffusion, across interfaces in WDM [77–80].

The development of new facilities such as free-electron lasers (FELs) in the extreme ultraviolet radiation (XUV) to hard X-ray range has revitalized research on WDM, allowing for the heating and probing of samples on ultrafast timescales of 1–100 fs, which is shorter than the inverse phonon frequency of solids. This opens up the possibility of conducting experiments that, rather than trying to achieve an equilibrated system, intentionally create a non-equilibrium state with hot electrons and cold ions in order to study non-equilibrium effects [81,82]. However, the novel sources provided by FELs not only provide an ability to isochorically heat systems to well-defined conditions, but are also an extremely bright source for scattering, imaging, and spectroscopic probing [83]. A particular improvement in diagnostic capability provided by FELs is in X-ray Thomson scattering, with some key advantages over the use of X-rays from laser plasmas. The enhanced collimation and monochromatic nature of the probe are extremely well suited to this diagnostic and this has allowed many advances. For example, XUV Thomson scattering experiments have been used to test electron–ion equilibration models on ultra-short timescales [84]. At the same time, X-ray scattering experiments have been able to probe the structure and dynamics of warm dense matter with unparalleled resolution [85], with measurements of the damped electron plasmon wave by Sperling *et al.* being used to show that the dynamic conductivity exhibits non-Drude-like behaviour that must be considered when determining the optical properties of WDM, rather than relying on the widely used random phase approximation [86].

In addition to transport and equilibration, another important aspect of the electronic properties of warm dense matter is the stopping power of charged particles, which is relevant to fields such as planetary science, geophysics and fusion research, where the stopping of alpha particles can have a significant impact on capsule performance. Experiments on stopping power in warm dense matter, such as those conducted by Malko *et al.* [87], have found deviations from classical models for protons in warm dense carbon but have shown better agreement with first-principles calculations using time-dependent DFT, as demonstrated by Ding *et al.* when applied to warm dense Be experiments performed by Zylstra *et al.* [88]. This time-dependent DFT approach showed a significant deviation from classical models when applied to alpha-particle stopping in warm dense DT plasmas relevant to fusion capsules. Angermeier *et al*. [89] will discuss the related issue of diffusion of ions in warm dense matter. This is important in the field of fusion pellets, where fuel mixing is a key factor in capsule performance.

## 5. Modelling approaches

This volume includes papers that discuss various numerical simulation techniques used in the field of WDM, and their recent advancements. As noted in the Introduction, sometimes approaches from liquid metal theory have been used. An example used to investigate the ion–ion structure is the use of the Ornstein–Zernicke equation and hypernetted chain closure relations (e.g. [90]), where a given interionic potential is used to develop a self-consistent solution to the pair-distribution function, g(r). This given potential might, for example, take the form of a screened Coulombic potential introduced by Wünsch *et al.* [91]. This potential included the addition of a stiff repulsive term to account for the overlap of bound shells as ions approach closely,

5.1 $$V(r)=\frac{A}{r^{4}}+\frac{Z^{2}\;e^{4}}{r}\;e^{\left(-r/\lambda \right)},$$

where the strength of the first term is determined by the parameter A, and there is a screening length, λ, which is typically set somewhere between the Debye and Thomas–Fermi screening lengths, depending on the degeneracy [92]. The HNC approach is practical and fast for an assumed steady state with a fixed, equilibrated temperature, a known interaction potential and moderate coupling [93]. However, this is not the case for many of the cases of interest here. For example, solids that are rapidly heated, such as those produced by FEL sources, may experience rapid changes in temperature as the energy exchange between electrons and ions takes place. This can result in both thermal melting, in which the ions gain enough energy to reach the melting point at the given pressure, and non-thermal melting, in which the ejection of electrons through photo-ionization and subsequent electron–electron collisions leads to a potential surface where the ions are no longer bound to each other. For more strongly coupled systems, it may be necessary to use other techniques, such as ab initio molecular dynamics, to understand the material behaviour and evolution.

Molecular dynamics is an important class of simulation techniques that can model a system of many particles with differing levels of physics assumptions made. A classical approach can be taken, with a predetermined pairwise potential to govern the forces between ions, in a similar way to the HNC approach above. Historically, a Coulomb [94] or screened Coulomb potential [95] was used. However, recent more advanced potentials have been developed, including short-range repulsion effects [91,96] and thermally damped Friedel oscillations [97]. These classical simulations have been used to investigate many WDM properties such as density fluctuations [96,98], transport coefficients [99,100] and electron–ion equilibration [101]. Molecular dynamics is able to capture the strongly coupled nature of warm dense matter, but the quantum effects are hidden in the details of the interatomic potential that ultimately defines the accuracy of these simulations. In many cases, the parameters and even the form of the potential are unknown. In such cases, we must use more advanced techniques, such as density functional theory (DFT), to calculate the potential. As an example of the power of the molecular dynamics approach, Ling *et al.* [102] used molecular dynamics to simulate the formation of warm dense Cu using the shock compression method, and find the complicated evolution of the thermodynamic paths during the compressing processes.

DFT is a popular simulation technique in the WDM field, with DFT molecular dynamics (DFT-MD) a popular method of choice. The theory is based on the concept that the total energy is a function of the electron density distribution, and by minimizing this energy, we can determine the actual electron distribution. The Thomas–Fermi model is often seen as a precursor to DFT, but it was Hohenberg & Kohn’s [103] and Kohn & Sham’s [104] work that truly advanced the field. For more information about DFT, see Kohanoff’s work [105]. One challenge for DFT is properly formulating expressions for exchange and correlation energies in non-uniform systems. While DFT is commonly used in solid-state physics, it can be computationally intensive for warm dense matter at high temperatures due to the need to include a large number of excited state orbitals in the simulation, which scales cubically with the number of orbitals. Using DFT, Mazevet *et al.* [106] have studied the time evolution of a sample of gold assuming an initial, essentially instantaneous, input of energy to the electrons. Using a combination of classical molecular dynamics with ab initio methods, where the forces acting are derived in a time-dependent way, governed by the configuration of the ions at a particular point in the simulation, they were able to follow the melting of the lattice and equilibration of the ion and electron temperatures.

These ab initio methods are well suited to the study of time-dependent problems [107], in which the electronic states and orbitals are calculated from the atomic structure, which then determines the forces that move the particles—essentially, using DFT to calculate the potential for MD on-the-fly. With increased computing power, such simulations have become possible over the last decade, with DFT-MD being used to simulate the dynamic properties of WDM with increasing levels of complexity [108–110]. Of course, there have also been significant developments in the method. White & Collins [111] developed algorithms that allow temperatures in the 50 eV range to be handled with acceptable computing times while considerable effort is now being spent on the development of high-temperature exchange and correlation functionals [112]. With the increased interest in machine learning, the accurate interatomic potentials from DFT data can be produced for WDM, so that we can extend the study of WDM to millions of atoms within ab initio accuracy [113,114].

Incorporating electronic quantum effects into first-principles simulations is inherently computationally demanding, limiting their applicability to larger spatiotemporal scales and non-equilibrium systems; one way around this is to use reduced models. Quantum hydrodynamics, which has recently gained renewed interest [115], is able to include the important role of quantum effects in cases where the quantum correlations are not too prominent [116] and consider the role of quantum non-locality through the inclusion of the Bohm potential, which is neglected in classical hydrodynamics. It is noteworthy that in certain scenarios, the Bohm force arising from this approach can be comparable in magnitude to the forces generated by exchange and correlation pressures, highlighting the relevance of quantum hydrodynamics in capturing essential quantum phenomena [117].

A further important development has been wave packet molecular dynamics (WPMD). This time-dependent quantum mechanical method simultaneously simulates the classical point particle motion of ions and the quantum-mechanical behaviour of electrons [118]. It has become popular in recent years as a computationally fast way to study dynamic processes in warm dense matter that go beyond the Born–Oppenheimer approximation [119,120]. Such non-adiabatic techniques are necessary for describing states that are not in local thermal equilibrium ($T_{e}\neq T_{i}$), in which energy can be exchanged between electrons and ions through collisions [121]. However, recent research has indicated that these techniques may also be important for studying systems in equilibrium [122,123]. Angermeier *et al.* [89] investigate the role non-adiabatic effects have in particle diffusion in warm dense hydrogen, while Svensson *et al.* [124] present an extension of the wave packet description of quantum plasmas, in which non-isotropic wave packets can be elongated in arbitrary directions.

As we have noted, molecular dynamics methods, particularly those based on DFT, have been de-rigeur in warm dense matter modelling. However, we should not ignore the potential contributions of other methods. For example, Filinov *et al.* [125] present ab initio calculations of helium based on path integral Monte-Carlo methods that could be applied across a range of conditions, including WDM.

The evolution of the microscopic structure of warm dense matter in rapidly evolving systems is clearly a key interest for this volume. However, the calculation of transport coefficients, which describe the rate at which key properties are transferred throughout the plasmas, is another important category of dynamic processes. Transport properties such as thermal conductivity, viscosity and diffusion are some most fundamental dynamical parameters that reflect the nature of the interatomic potential and characterize the thermodynamics of the system. The corresponding transport coefficients, which encapsulate the rate of transfer of heat, momentum and mass, respectively, are a necessary input for continuum models such as magnetohydrodynamics, and all three coefficients play a vital role in our understanding of planetary interiors and the design and analysis of inertial confinement fusion (ICF) implosions. As highlighted at a recent workshop [126], there still exist large order-of-magnitude differences between theoretical predictions.

Several authors have carried out extensive theoretical discussions of electron-ion relaxation times for dense plasmas and warm dense matter (e.g. [127]). Dharma-Wardana & Perrot [128] used a Fermi Golden Rule (FGR) approach to calculate the interaction between the normal modes of independent ion and electron subsystems. Depending on whether they assumed the subsystems to have independent spectra or a spectrum of modes generated by the interaction between the subsystems, they found for solid density Al at temperatures in the range 3–40 eV, the coupling rate was 4 or 5 orders of magnitude lower than calculated with the classical Spitzer–Brysk method. For systems where the ion acoustic or phonon modes interact strongly with the electrons, coupled modes may form, further reducing the equilibration rate and forming a relaxation bottleneck [39,128]. Gericke *et al.* [129] investigated the validity of ad-hoc approximations to the Coulomb logarithm that are designed to extend a classical Landau–Spitzer approach to strongly coupled systems where linear approximations to the electron trajectory are not valid and both close and weak collisions are included.

In highly non-equilibrium systems undergoing rapid changes, such as in laser-excited solids, using FGR is thought to overestimate the transition rates on short time scales [130,131]. Models based on the extension of FGR, such as those based on the Eliashberg spectral function formalism [132], are thought to overestimate the coupling strength compared to experimental data. The most successful descriptions take into account the varying coupling between electrons and different phonon modes [133], with a hybrid approach based on tight-binding molecular dynamics code showing good agreement with the limited experimental data [134]. In this volume, the issue of electron–ion equilibration is addressed further in the paper of Ziaja *et al.* [135].

## 6. Conclusion

It is clear that warm dense matter is a field that is of wide interest for applications in fusion sciences, geophysics and planetary sciences. It is also a vibrant field with many advances, both in experimental techniques, computational simulation and underlying theory. These advances are exemplified by the papers presented and the works that they build upon and reference. The literature on warm dense matter is wide enough and varied enough that even within the scope of this special edition, concentrating on issues of time dependence and dynamic processes, we can see a huge variety of techniques and topics addressed.

## Data Availability

This article has no additional data.
